# Accelerating silage maize breeding: forage yield and nutritive quality of doubled haploid-derived hybrids

**DOI:** 10.7717/peerj.20197

**Published:** 2025-10-17

**Authors:** Sinem Zere Taşkın, Ahmet Duman, Uğur Bilgili

**Affiliations:** 1 Department of Field Crops, Bursa Uludag University, Bursa, Türkiye; 2 Maize Research Institute, Sakarya, Türkiye

**Keywords:** *Zea mays*, Forage yield, Forage quality

## Abstract

**Background:**

Maize (*Zea mays* L.) is a widely cultivated cereal crop for silage production due to its high biomass yield and nutritional value. Developing high-yielding and nutritionally superior hybrids is increasingly vital in the face of climate change and rising global forage demand. Doubled haploid (DH) technology accelerates hybrid development by enabling the rapid production of fully homozygous pure lines. This study aimed to evaluate silage maize hybrids derived from DH inbred lines for the effects of yield and nutritional quality.

**Methods:**

Eight recently developed hybrids (derived from doubled haploid inbred lines: S1, S2, S6, S9, and S11; derived from conventional inbred lines: S7, S8, and S10) and four commercial silage checks (S3, S4, S5, and S12) were used in this study. The experiments were arranged in a randomized complete block design with three replications. In this study, agronomic traits including plant height, ear ratio, stem ratio, and forage yield were evaluated, along with forage quality parameters such as dry matter, crude protein, neutral detergent fiber (NDF), acid detergent fiber (ADF), acid detergent lignin (ADL), and relative feed value.

**Results:**

Genotypes S6 and S9 provided higher forage yield, plant height, ear ratio, and a lower stem ratio. All genotypes of dry matter, crude protein, NDF, and ADF values were within the desired range. DH-derived hybrids outperformed checks and those derived from conventional inbred lines regarding forage yield and the investigated agronomic traits. The DH technology has great potential in developing high-yielding hybrids in a short time in silage maize breeding.

## Introduction

Maize (*Zea mays* L.) is the most extensively cultivated cereal crop worldwide and plays a critical role in livestock nutrition, not only as grain but particularly as whole-plant silage due to its high biomass yield and nutritional value. According to the Consultative Group for International Agricultural Research (Consultative Group on International Agricultural Research (CGIAR), 2025), maize demand in developing countries is projected to double by 2050. In Türkiye, green forage production reached 38.3 million tons in 2022, with silage maize accounting for 28.6 million tons, approximately 42% of total forage production (TUIK, 2023). The growing global population and the impacts of climate change underscore the urgent need to develop high-yielding and climate-resilient maize varieties, especially for silage purposes.

Maize is widely used as a silage crop both globally and in Türkiye due to its high dry matter yield per unit area, superior energy content, compatibility with mechanized harvesting, ease of ration formulation, and high palatability for dairy cattle (Fernandez et al., 2004). Successful silage maize production relies on proper variety selection and timely agronomic practices (Oliveira et al., 2019). The selection of a suitable hybrid in silage production is important because each cultivar tends to show different agronomic and nutritional responses depending on its degree of adaptation to the growing region conditions (Zopollatto et al., 2009). For commercial cultivars or hybrids, yield performance is closely linked to their stability across different environments (Eberhart & Russell, 1966). Therefore, environmental stability has an important criterion in maize breeders (Oliveira et al., 2014; Greveniotis et al., 2021), and breeders aim to develop genotypes that can keep high yield potential while being adapted to many environments or less affected by environmental changes.

Despite its importance, most commercially available silage maize varieties are selected primarily for high green forage yield, with limited focus on quality traits. Özata, Öz & Kapar (2012) stated that due to the limited number of cultivars officially registered for silage purposes, the majority of the seed demand for silage maize hybrid production is supplied by non-silage varieties in Türkiye; however, in recent years, high-yielding and good-quality silage maize cultivars have been developed and registered.

The first stage in maize breeding programs is the development of homozygous inbred lines that will be parents of hybrid varieties (Cerit et al., 2016). Then, crosses are made between the homozygous materials. However, conventional plant breeding is time-consuming and demands significant labor and financial resources. Achieving inbred lines through the classical self-pollination method takes six to eight generations; even then, about 98% homozygosity can be attained. In contrast, the DH technology can produce 100% homozygous inbred lines quickly. DH technology is widely utilized in current maize breeding to create homozygous parental lines for maize breeding and generate DH populations due to its high genetic variance and time-saving advantages. This technology significantly reduces the time required to achieve homozygous lines and offers benefits such as lower labor and financial resource requirements (Geiger & Gordillo, 2009; Chaikam et al., 2019).

Previous studies have shown that DH-derived test hybrids can outperform conventional hybrids in terms of grain yield and agronomic performance (Sserumaga et al., 2016, 2018; Wang et al., 2019). Identifying the optimal combination of inbred lines in hybrid breeding is crucial for developing high-performing hybrids. Additionally, evaluating the agronomic performance of hybrids derived from DH lines is important (Sadessa et al., 2024).

This study aimed to evaluate the forage yield, yield components, and nutritional quality of silage maize hybrids developed from DH lines, and to compare their performance with those derived from conventional inbred lines and commercial cultivars.

## Materials and Methods

### Plant materials

Eight recently developed hybrids (derived from DH: S1, S2, S6, S9, and S11; derived from conventional inbred lines: S7, S8, and S10) and four commercial high-yielding silage checks (S3: Dekalp 6442, S4: Dekalp 6777, S5: Dekalp 7240, and S12: Pioneer 31Y43) were used in this study. Among the genotypes used in the experiment, S1, S2, S4, S11, and S12 belong to the FAO 650 maturity group; S3 to the FAO 630 maturity group; S5 to the FAO 700 maturity group; and S6, S7, S8, S9, and S10 to the FAO 720 maturity group. The following sections outline the breeding procedures used to generate both DH-derived and conventionally developed hybrids evaluated in this study.

Heterotic groups were determined by the top-cross method from selfed lines (generation S4–S5) developed *via* classical breeding methods (pedigree system). Two widely used public tester lines were employed to determine the heterotic groups of the lines, one belonging to the Lancaster (FRMo 17) and the other to the Stiff Stalk (FRB 73) heterotic group. Based on heterotic grouping and top-cross performance, selected lines were advanced to the S6–S7 generations through continued selfing. In the top-cross trial, grain yield was considered the primary parameter for predicting heterotic groups. Progress in breeding was achieved by taking both specific combining ability (SCA) and general combining ability (GCA) into account. In the first year, 200 hybrid candidates were evaluated in yield and adaptation trials across four locations. The inbred lines were selected from preliminary yield trials according to their adaptive traits to silage, such as long plant height, resistance to lodging, high total plant yield, high ear yield, and a high leaf/stem ratio. As a result, 40 promising hybrids were predicted. In the second year, preliminary yield trials were repeated, and a second selection was conducted. As a result, three hybrids (S7, S8, and S10) were selected for inclusion in the registration trial.

DH lines were developed using the *in vivo* maternal haploid method, as described by Röber, Gordillo & Geiger (2005) and Chaikam et al. (2019). Donor genotypes with FAO maturity groups ranging from 500–750 were crossed with the Stock6 inducer line. Haploid seeds were obtained after hybridization, germinated at 23 °C in a dark climate chamber, and treated with a solution containing 0.06% colchicine and 0.5% dimethyl sulphoxide at 18 °C for 12 h (Gayen et al., 1994; Deimling, Röber & Geiger, 1997). Fertile DH plants were self-pollinated to produce inbred lines. These DH lines were subjected to preliminary yield trials at four locations. As with the conventionally bred lines, selection was based on morphological traits and quality characteristics. In the first year, 15 out of 20 initial candidates were advanced, based on silage-relevant traits such as tall plant height, lodging resistance, high total and ear yield, and a favorable leaf/stem ratio. Following the second-year trials, five DH-derived hybrids (S1, S2, S6, S9, and S11) were selected for further evaluation.

### Research locations

The field experiments were conducted at Bursa (40°11′N, 29°04′E, and altitude 100 m), Türkiye, in 2021–2022. Based on the Köppen–Geiger climate classification system, which considers the average temperatures of the hottest and coldest months, the Bursa region is classified as Csa, indicating a hot-summer Mediterranean climate with dry summers and mild, wet winters. In contrast, Sakarya falls under the Cfa category, representing a humid subtropical climate with hot summers and year-round precipitation (Yılmaz & Çiçek, 2018).

The weather data are presented in Table 1. During the growing season (May–October), average temperatures at both locations were comparable to the long-term average of 21.2 °C. In Bursa, average humidity levels exceeded the long-term average of 65.4%, while total precipitation was considerably lower, amounting to 257.1 mm. In contrast, Sakarya experienced higher total precipitation than its long-term average of 382.9 mm in first year, but lower in second year. Average humidity values in Sakarya remained below the long-term average of 72.3% in both years. Because precipitation in Sakarya was evenly distributed throughout the growing season, no irrigation was applied in the trials conducted there. Conversely, a drip irrigation system was installed for the Bursa trials, and irrigation was carried out regularly due to inadequate rainfall.

**Table 1:** Monthly climate data (temperature, precipitation, and relative humidity) for Sakarya and Bursa research locations during the 2021 and 2022 growing seasons, along with long-term averages (1990–2020).

Location	Years	Months						Total/ Average
		May	June	July	August	Sept.	Oct.	
**Temperature (°C)**								
Sakarya	2021	19.0	21.0	25.2	25.1	19.6	15.0	20.8
	2022	18.2	22.7	23.8	25.5	21.3	16.0	21.3
	LT*	17.9	22.1	24.3	24.4	20.6	16.4	21.0
Bursa	2021	18.6	20.9	25.5	25.9	20.3	14.7	21.0
	2022	17.8	22.3	24.0	25.3	20.7	15.8	21.0
	LT*	18.0	22.5	25.1	25.1	20.8	15.9	21.2
**Precipitation (mm)**								
Sakarya	2021	74.4	88.1	101.5	55.8	81.9	43.2	444.9
	2022	34.8	112.1	10.5	61.7	26.8	77.3	323.2
	LT*	59.3	84.8	49.7	50.9	53.0	85.2	382.9
Bursa	2021	14.5	61.7	32.8	0.1	10.9	42.0	162.0
	2022	16.4	129.2	2.1	40.3	39.2	11.7	238.9
	LT*	48.5	42.1	15.1	16.9	50.3	84.2	257.1
**Relative humidity (%)**								
Sakarya	2021	62.9	73.0	72.5	69.5	72.1	78.3	71.4
	2022	62.2	71.8	68.7	74.0	66.5	78.9	70.4
	LT*	71.3	69.6	70.8	72.2	73.6	76.7	72.3
Bursa	2021	67.1	73.0	66.1	60.6	64.5	72.8	67.4
	2022	71.2	69.9	63.8	63.9	66.7	71.0	67.7
	LT*	66.9	62.7	59.4	61.3	67.4	74.7	65.4

**Note:**

* LT, Long term (1990–2020).

Soil properties are presented in Table 2. Total nitrogen was determined using the Kjeldahl method, total phosphorus by colorimetry with a PG Instruments T60 UV/VIS spectrophotometer, and total potassium with an Eppendorf Elex 6361 flame photometer after acid digestion with HNO₃. Organic carbon was measured by the Walkley–Black method. Soil pH and EC were determined in a 1:5 soil-to-ultrapure water suspension (w/v). According to the analysis result, the soils of both locations had a clay-loam structure, were poor in nitrogen and organic matter, and had no salinity problem.

**Table 2:** Physical and chemical soil properties of the experimental sites in Sakarya and Bursa, including pH, electrical conductivity (EC), texture, nitrogen, organic matter, phosphorus (P_2_O_5_), and potassium (K_2_O) content.

Locations	pH	EC (mS m^−1^)	Texture class	*N* (%)	Organic matter (%)	P_2_O_5_ (kg ha^−1^)	K_2_O (kg ha^−1^)
Bursa	7.9	0.351	clay-loam	0.069	1.35	39.8	4,371
Sakarya	7.5	0.017	clay-loam	0.060	1.19	138.8	1,790

### Experimental design

The experiments were arranged in a randomized complete block design with three replications. The experimental plot size was 273 m^−2^ (two rows, each 5 m long). Distances between and within rows were 0.70 and 0.15 m, respectively. Experiments were established in May in both years and locations. In both locations, NPK (20:20:0) fertilizer was applied as a basal fertilizer at a rate of 50 kg da^−1^. When the plants reached a height of 40–50 cm, nitrogen fertilizer (urea, 46% N) was applied at a rate of 40 kg da^−1^.

### Measurement

In this study, forage yield (t ha^−1^), some yield components (plant height (cm), ear ratio (%), stem ratio (%), dry matter (%)), crude protein (%), neutral detergent fiber (% NDF), acid detergent fiber (% ADF), acid detergent lignin (% ADL) and relative feed value (RFV) parameters were measured. Forage yield was determined by harvesting two rows and converted into t per ha. Ten plants in the middle of each plot were used to determine the following agronomic parameters: plant height, ear ratio, and stem ratio. Plant samples taken from each genotype were dried in an oven at 75 °C for 24 h (Hatami et al., 2022). Before chemical analyses, the dried plant samples were ground in a mill with a sieve diameter of 1 mm. Nitrogen contents of the genotypes were determined by the Kjeldahl method, and these values were multiplied by the coefficient of 6.25 to obtain CP ratios (AOAC, 2023). NDF, ADF, and ADL contents were determined on ANKOM200 Fiber Analyzers (ANKOM Technology Corporation, Fairport, NY, USA) according to the methods specified by ANKOM Technology (2023). The following equations were used to calculate dry matter intake (DMI), digestibility (DDM), and relative feed value (RFV) (Grant et al., 2014):

${\rm \% DDM = 88.9 - (0.779 * \% ADF \, dry\, matter\, basis)}$

${\rm DMI \% = 120/ \%NDF \,dry\, matter\, basis}$

${\rm RFV = (\%DDM * \%DMI)/1.29}.$

### Statistical analyses

All collected data were subjected to analysis of variance (ANOVA) based on a randomized complete block design (RCBD). Genotypic differences were evaluated using F-tests at the 0.05 and 0.01 significance levels. When significant differences were observed, means were separated using Fisher’s protected least significant difference (LSD) test. All statistical analyses were performed using JMP® Pro 13 software (SAS Institute Inc., Cary, NC, USA).

## Results

The main effects of year (Y), location (L), genotype (G), and their interactions were significant at the 1% significance level for all traits except for stem ratio and forage yield in the L and Y × L. Considering our findings, the performance of genotypes varied for the studied traits across each location and year (Table 3).

**Table 3:** Analysis of variance (ANOVA) results for plant height, ear ratio, stem ratio, and forage yield, showing the effects of year, location, genotype, and their interactions.

Source	DF	Plant height	Ear ratio	Stem ratio	Forage yield
Year (Y)	1	42,025.0**	126.5**	61.3**	10,627,848**
Location (L)	1	1,406.3**	16.6**	0.6	334,861
Y × L	1	5,256.3**	16.6**	0.6	412,406
Block	8	847.2	3.3	25.5**	2,763,825**
Genotypes (G)	11	22,259.7**	353.0**	535.3**	52,085,554**
G × Y	11	21,879.1**	165.1**	149.9**	11,742,011**
G × L	11	3,214.5**	93.4**	51.9**	15,132,519**
G × Y × L	11	12,339.5**	93.4**	51.9**	12,002,267**
Error	88	8,786.1	68.6	101.7	10,349,189

**Notes:**

DF, Degree of Freedom.

**: significant at *p* < 0.01.

The mean values of results investigated over years and locations of plant height, ear ratio, stem ratio, and forage yield were found between 311–396 cm, 28.3–39.3%, 26.0–38.6%, 65.2–100.9 t per ha, respectively (Table 4).

**Table 4:** Mean values of plant height (cm), ear ratio (%), stem ratio (%), and forage yield (t ha^−1^) for twelve maize genotypes in two locations during the experimental period.

Genotypes	Plant height (cm)					Ear ratio (%)				
	Bursa		Sakarya		Genotype mean	Bursa		Sakarya		Genotype mean
	Year I	Year II	Year I	Year II		Year I	Year II	Year I	Year II	
S1	341 ab	346 d	308 def	371 c	342 cde	34.0 e–h	37.0 bcd	34.3 cd	37.0 b	35.5 c
S2	331 bcd	340 d	336 ab	371 c	345 bcd	35.3 bcd	35.6 def	35.6 bc	35.3 c	35.5 c
S3	296 g	326 ef	356 a	310 e	322 f	35.6 bc	34.6 ef	36.6 ab	35.0 cd	35.4 c
S4	296 g	316 f	315 c–f	320 e	312 g	34.6 c–f	37.6 abc	33.0 de	37.6 ab	35.7 c
S5	343 ab	338 de	323 bcd	353 d	339 de	33.3 gh	31.6 g	29.0 f	32.3 e	31.4 f
S6	355 a	378 a	315 c–f	396 a	361 a	38.0 a	39.3 a	35.6 bc	38.6 a	38.1 a
S7	331 bcd	350 cd	311 c–f	381 abc	343 cd	35.3 bcd	36.3 cde	28.3 f	36.6 b	34.1 de
S8	311 efg	345 d	310 c–f	370 c	334 e	36.0 b	38.6 ab	29.6 f	38.3 a	35.8 c
S9	323 cde	368 ab	330 bc	390 ab	352 b	33.6 fgh	37.6 abc	37.6 a	37.0 b	36.6 b
S10	306 fg	368 ab	295 f	390 ab	340 cde	33.0 h	34.3 f	33.6 d	34.0 d	33.8 e
S11	336 bc	360 bc	320 b–e	376 bc	348 bc	35.0 b–e	35.6 def	32.0 e	35.3 c	34.5 d
S12	316 def	316 f	300 ef	345 d	319 fg	34.3 d–g	34.0 f	36.3 ab	34.3 cd	34.6 d
LSD_*(p ≤ 0.05)*_	16.1	12.7	21.3	16.3	8.1	1.1	1.6	1.3	1.6	0.7
Year mean	324 c	346 b	318 d	364 a		34.8 b	36.1 a	33.5 c	35.9 a	
Location mean	335 b		341 a			35.4 a		34.7 b		
	**Stem ratio (%)**					**Forage yield (t ha** ^**−1**^ **)**				
S1	32.6 de	35.3 cd	34.6 abc	35.3 cd	34.5 bcd	95.3 bc	87.6 abc	85.5 e	90.0 bc	89.6 c
S2	35.3 ab	34.6cd	36.6 a	34.6 cd	35.3 ab	94.8 bc	86.6 bcd	95.8 abc	86.4 cde	91.9 bc
S3	33.6 cd	34.6 d	34.3 bcd	34.6 cd	34.3 cd	76.0 f	80.4 ef	92.0 cd	73.1 h	80.4 e
S4	34.3 bc	35.6 bcd	32.6 cde	35.6 bcd	34.5 bcd	89.3 d	78.5 ef	72.5 f	74.6 gh	78.7 e
S5	26.0 g	29.3 g	31.3 ef	29.3 g	29.0 g	93.0 cd	83.9 cde	95.0 abc	86.0 cde	89.5 c
S6	32.6 de	36.3 bc	30.0 f	36.3 bc	33.8 d	100.3 a	91.3 ab	98.6 ab	100.7 a	97.7 a
S7	30.0 f	32.0 f	32.6 cde	32.0 f	31.6 f	79.4 ef	77.0 f	86.7 de	80.2 efg	81.8 e
S8	31.3 ef	32.6 ef	33.6 bcd	32.6 ef	32.5 e	93.8 cd	92.4 a	65.2 g	87.1 cd	84.6 d
S9	35.0 abc	34.0 de	33.6 bcd	34.0 de	34.2 cd	100.9 a	90.9 ab	100.0 a	96.1 ab	97.0 a
S10	36.0 a	34.3 de	35.3 ab	34.3 de	35.0 bc	92.8 cd	80.6 ef	91.7 cde	76.8 fgh	85.5 d
S11	36.0 a	37.3 ab	33.6 bcd	37.3 ab	36.1 a	98.7 ab	89.2 abc	97.9 abc	86.6 cde	93.1 b
S12	34.6 abc	38.6 a	32.3 de	38.6 a	36.1 a	83.5e	81.2 def	92.5 bcd	83.4 def	85.15 d
LSD_*(p ≤ 0.05)*_	1.3	1.7	2.2	1.7	0.8	459.0	552.9	631.2	658.6	278.2
Year mean	33.1	34.5	33.4	34.5		91.5	85.0	89.4	85.1	
Location mean	33.8		34.0			88.2		87.3		

**Notes:**

Small letters in the same column indicate a significant difference according to least significant difference.

At the Bursa location, the shortest plants were observed in genotypes S3 (296 cm) and S4 (296 cm) in the first year, and in S4 (316 cm) and S12 (316 cm) in the second year. The tallest plants were recorded in S6 (355 cm in the first year, 378 cm in the second year) across both years. Significant variation was also observed in ear and stem ratios. Genotype S6 (38.0% in the first year, 39.3% in the second year) consistently showed the highest ear ratio across years. Regarding stem ratio, genotypes S10 (36.0%) and S11 (36.0%) exhibited the highest values in the first year, whereas S12 (38.6%) had the highest value in the second year. In contrast, S5 (26.0% and 29.3% in the first and second years, respectively) displayed the lowest stem ratio in both years. Genotypes S6 (100.3 t ha^−1^) and S9 (100.9 t ha^−1^) had higher forage yields in the first year, while S8 (92.4 t ha^−1^) exhibited the highest forage yield in the second year (Table 4).

At the Sakarya location, genotype S3 (356 cm) exhibited the greatest plant height in the first year, whereas S6 (396 cm) was the tallest in the second year. The lowest plant height was recorded in genotype S10 (295 cm) in the first year, and in genotypes S3 (310 cm) and S4 (320 cm) in the second year. The highest ear ratio was obtained from genotype S9 (37.6%) in the first year, and from genotypes S6 (38.6%) and S8 (38.3%) in the second year. The lowest ear ratio values were observed in genotypes S5 (29.0%), S7 (28.3%), and S8 (29.6%) in the first year, and in genotype S5 (32.3%) in the second year. Regarding stem ratio, S2 (36.6%) recorded the highest value in the first year, while S12 (38.6%) stood out in the second year. The lowest stem ratio was recorded in S6 (30.0%) in the first year, and in S5 (29.3%) in the second year. Similar to the Bursa location, genotypes S6 (98.6 and 100.7 t ha^−1^ in the first and second years, respectively) and S9 (100.0 and 96.1 t ha^−1^ in the first and second years, respectively) produced the highest forage yields in Sakarya. On the other hand, genotype S8 (65.2 t ha^−1^) in the first year and S3 (73.1 t ha^−1^) in the second year were identified as the lowest-yielding genotypes (Table 4).

Based on genotype mean values, the tallest plants were observed in genotype S6 (361 cm), indicating superior vegetative growth. The shortest plants were recorded in genotypes S3 (322 cm) and S4 (312 cm). The highest ear ratio was found in genotype S6 (38.1%). The lowest values were observed in genotype S5 (31.4%), followed by S10 (33.8%). The highest stem ratios were recorded in genotypes S11 and S12 (both 36.1%), and S10 (35.0%). The lowest stem ratio was observed in genotype S5 (29.0%), followed by S7 (31.6%). The highest forage yield was obtained from genotypes S6 (97.7 t ha^−1^) and S9 (97.0 t ha^−1^), reflecting their high productivity potential. The lowest forage yields were observed in genotypes S3 (80.4 t ha^−1^) and S4 (78.7 t ha^−1^).

Statistically significant differences were found in the year and location means for plant height and ear ratio. However, stem ratio and forage yield were not statistically significant, concerning locations and year means. The highest year and location means for plant height was recorded in second year at the Sakarya location. For the year mean, high ear ratio values were recorded in second year at both locations. The Bursa location produced the highest location mean value for the ear ratio (Table 4).

The chemical analysis results are presented in Table 5. Except for dry matter (DM) content, statistically significant differences were observed among genotypes for all evaluated parameters, including crude protein (CP), neutral detergent fiber (NDF), acid detergent fiber (ADF), acid detergent lignin (ADL), and relative feed value (RFV). All parameters, except DM, were statistically significant at a 1% significance level (Table 5).

**Table 5:** ANOVA results for dry matter (DM), crude protein (CP), neutral detergent fiber (NDF), acid detergent fiber (ADF), acid detergent lignin (ADL), and relative feed value (RFV) across maize genotypes.

Sources of variation	DF	DM (%)	CP (%)	NDF (%)	ADF (%)	ADL (%)	RFV
Genotypes	11	ns	4.143**	562.60**	45.82**	6.04**	5,004.8**
Block	2	ns	ns	ns	4.78*	ns	ns
Error	22	26.8	0.81	26.6	12.0	1.41	304.4

**Notes:**

ns, not significant.

*, **: significant at *p* ≤ 0.05 and *p* ≤ 0.01, respectively.

Dry matter content ranged from 32.3% (S5) to 35.7% (S6), while CP values varied between 8.6% (S5) and 9.7% (S6). NDF content was lowest in S6 (41.4%) and highest in S5 (55.8%). ADF values ranged from 24.3% in S1 to 28.0% in S12. ADL content showed variation between 1.8% (S11) and 3.3% (S5). RFV was highest in S6 (155.7) and lowest in S5 (112.1) (Table 6).

**Table 6:** Mean values of forage quality parameters DM, CP, NDF, ADF, ADL, and RFV for the twelve maize genotypes.

Genotypes	DM	CP (%)	NDF (%)	ADF (%)	ADL (%)	RFV
S1	33.9	8.9 e–g	44.0 f	24.3 e	2.4 cd	147.7 b
S2	33.5	9.3 b–d	49.9 c	26.4 cd	2.6 b–d	127.3 ef
S3	32.9	9.6 ab	53.6 b	26.3 cd	2.9 ab	118.6 g
S4	32.5	9.5 a–c	49.9 c	26.8 bc	3.0 ab	126.7 ef
S5	32.3	8.6 g	55.8 a	27.7 ab	3.3 a	112.1 h
S6	35.7	9.7 a	41.4 g	25.0 e	2.4 cd	155.7 a
S7	33.4	8.7 fg	50.4 c	27.3 a–c	2.3 d	124.8 fg
S8	33.4	9.0 ef	48.6 c	25.4 de	2.5 cd	132.1 de
S9	32.9	9.2 c–e	45.9 de	27.2 a–c	2.7 bc	137.2 cd
S10	33.5	9.1 d–f	46.7 d	27.7 ab	2.9 ab	134.1 d
S11	32.9	8.8 fg	44.1 ef	27.4 a–c	1.8 e	142.4 bc
S12	32.9	8.9 fg	49.6 c	28.0 a	3.2 a	125.7 f
LSD_0.05_	ns	0.325	1.864	1.254	0.429	6.299

**Note:**

DM, Dry matter; CP, Crude protein; NDF, Neutral detergent fiber; ADF, Acid detergent fiber; ADL, Acid detergent lignin; RFV, Relative feed value; ns, not significant. Small letters in the same column indicate a significant difference according to the least significant difference.

## Discussion

According to the results of our study, the twelve maize genotypes differed significantly in terms of the measured traits. Generally, the high-yielding genotypes also showed high mean values in other agronomical characteristics.

Silage maize varieties are desired to have a high yield, a high leaf ratio, and a high ear ratio, but a low stem ratio (Güneş & Öner, 2019). The research results revealed that the highest forage yield was obtained from genotype S6, which was derived from DH lines. The S6 genotype also stood out due to its high ear ratio and low stem ratio. After S6, the S9 genotype was identified as the second most valuable genotype in terms of forage yield (97.0 t ha^−1^), ear ratio (36.6%), stem ratio (34.2%), and plant height (352 cm). Silage maize genotypes with a high ear ratio generally provide higher forage yield and quality, as ears make a substantial contribution to dry matter and energy content. A lower stem ratio is considered desirable in silage maize, as it generally corresponds to reduced lignin content in the plant tissue, thereby enhancing digestibility and improving forage quality (Karnatam et al., 2023). Hence, the S6 genotype, which combines a high ear ratio with a low stem proportion, can be expected to deliver both higher yield potential and improved nutritional quality. In addition, traits such as strong stems with resistance to lodging, low ear placement (ear height ratio ≤0.5), upright leaves above the ear and more horizontal leaves below, stay-green characteristics that prolong photosynthesis, and a robust root system are also important, and it should not be overlooked that these traits are particularly valuable in silage maize (Karnatam et al., 2023). These criteria were likewise taken into consideration during the breeding process of the genotypes investigated in our study.

In Sakarya, the yield in second year was lower than in first year, despite the taller plant heights. This discrepancy may be attributed to insufficient rainfall during the generative phase of the second year growing season (Tables 1 and 4). Maize is highly dependent on climatic factors such as rainfall and temperature (Li et al., 2019). Inadequate irrigation and rainfall can result in plant mortality or reduced yields (Omoyo, Wakhungu & Oteng’I, 2015). Baffour-Ata et al. (2021) supported this, reporting that significant changes in precipitation and temperature can lead to substantial variations in maize yield.

Linn et al. (2006), reported that ideal silage maize quality should range between 32–38% for DM, 8.0–9.5% for CP, 32.3–55.8% for NDF (desired range <41%), 18.3–34.9% for ADF (desired range <24%), and 2.1–5.4% for ADL (desired range <3.5%). Our findings indicate that all genotypes were within the range for DM (32.3–35.7), CP (8.6–9.7%), NDF (41.4–55.8%), and ADF (24.3–28.0%). However, ADL (1.8–3.3%) content was within the desired range for all genotypes except S11 (1.8%).

CP content is an important quality factor in silage maize, and this content should be high. This range is crucial for meeting livestock’s nutritional needs and maintaining the silage’s optimal nutritional value. In this study, the genotypes’ CP contents differed, and the highest value belonged to genotype S6. Although CP content is largely genetically controlled, it can be influenced by climate, planting and harvest times, cultivation techniques, and fertilization (Burgu & Mut, 2023). The observed differences among the genotypes may be attributable to these factors.

ADF refers to the amount of cellulose, lignin, and insoluble protein in the plant cell wall. ADF content is an indicator that gives an idea about the animal’s energy intake and feed digestibility. NDF is an expression of the amount of cellulose, hemicellulose, lignin, cutin, and insoluble protein in the plant cell wall and is a measure of the overall quality and digestibility of the feed. The NDF content directly impacts animal feed intake. The animal’s feed intake increases as the NDF and ADF content decreases (Bittman, 2004; Öktem, Öktem & Demir, 2021). In our study, genotype S6 exhibited the lowest NDF and ADF content, outperforming all control hybrids. Faria et al. (2021), in their evaluation of 323 silage maize samples collected between 2004 and 2015, reported average levels of 53.30% NDF, 32.02% ADF, and 4.25% ADL; these values are considerably higher than the mean values determined in the present study, which were 48.3% for NDF, 26.6% for ADF, and 2.6% for ADL.

Neumann et al. (2024) reported that ADL values in corn silage averaged 3.34% DM, with a wide range from 0.60% to 11.43%. The study demonstrated that higher ADL concentrations were strongly correlated with the indigestible carbohydrate fraction (C fraction), along with increased NDF and ADF contents, which in turn reduced total digestible nutrients (TDN), an estimate of forage energy value. These results highlight that elevated ADL decreases silage digestibility by enhancing cell wall lignification and limiting energy availability. Consequently, silages with higher ADL levels are likely to impair feed intake and animal performance due to reduced nutrient utilization efficiency. In our study, however, the ADL values of the genotypes were considerably lower than the average value (3.34%) reported by Neumann et al. (2024).

Given the variability in quality parameters, integrative measures such as RFV are often used (Grant et al., 2014; Zhang et al., 2018). According to Coppock (1997), RFV values above 151 are categorized as first-class (top quality), 151–125 as prime quality, 124–103 as good quality, 102–87 as medium quality, 86–75 as low quality, and below 75 as poor quality. In this study, S6 obtained the highest RFV, classifying it as ‘top quality’. All other genotypes, except the two check hybrids (S3 and S5), were categorized as prime quality.

The results indicate that the S6 genotype used in this study is a good candidate for the production of high-quality silage for ruminant nutrition. Also, generally, hybrids derived from DH inbred lines (S6, S9, S11) produced high forage yields and demonstrated better agronomic performance than the check hybrids and those derived from conventional inbred lines. These results were consistent with the results of other authors who reported that DH hybrids were superior compared to most commercial controls (Beyene et al., 2013, 2017). Similar observations were reported by Sserumaga et al. (2016) and Beyene et al. (2011) that doubled-haploid hybrids performed significantly better than commercial checks in their studies. Similarly, Sserumaga et al. (2018) stated that hybrids derived from DH inbred lines demonstrated higher performance in terms of grain yield and other agronomic traits than the commercial hybrids. The superior performance of DH-derived hybrids can be explained by the inherent advantages of DH technology. With DH technology, complete genetic homozygosity that normally requires 6–10 years in classical breeding can be achieved within a single generation. One advantage of haploids is that, since they carry only one copy of each gene, recessive mutations become visible. Haploid plants carrying harmful genes usually die, remain weak or sterile, and fail to produce seeds. In this way, unfavorable genes are rapidly eliminated at the haploid stage. This process is similar to natural selection, but it acts as a faster tool to remove harmful genes and increase favorable ones in the genetic pool. When the chromosomes of haploids are doubled, a DH line with 100% homozygosity is obtained. DH lines exhibit complete genetic uniformity and stability, which ensures phenotypic consistency in field trials and facilitates variety registration (Chang & Coe, 2009; Chaikam & Prasanna, 2020). Also, Hu et al. (2022) reported that DH lines are thought to be no different from conventional inbred lines; however, when it comes to test crossing, they differ in terms of the homozygosity levels and founder haplotype genomic compositions. Most DH lines display minimal recombination events, as DH lines are often generated from the F1 progeny of two parents with complementary advantageous phenotypes. Consequently, DH lines remain genetically more stable and homozygous. In our study, the fact that hybrids derived from DHs came to the forefront regarding agronomic performance and quality characteristics may be due to these reasons. However, it should be noted that DH technology may also present some limitations, such as restricted genetic diversity due to complete homozygosity, reduced recombination events, and variable success rates depending on species or genotype. In some cases, DH lines may also exhibit weak vigor or poor adaptability, which should be considered when integrating DH methods into breeding programs (Chaikam & Prasanna, 2020). Indeed, Vargas Escobar & García Dávila (2023), in their study evaluating fifteen DH lines and fourteen selfed lines (S2, S4, and S6) through multi-location yield trials, they reported that it took 3 years from the S1 population to identify DH lines with 100% homozygosity and the highest general combining ability (GCA), which was 1 year earlier than the S6 lines (98.4% homozygous). They also noted that DH lines exhibited stronger genotype × environment interactions than S2 testcrosses, but showed a heritability level comparable to that of the S6 generation.

In the regions where the study was conducted, Bursa and Sakarya, silage maize hybrids belonging mainly to the FAO 700 maturity group are generally preferred. Therefore, the genotypes S6 and S9, which stood out in our study, belong to the FAO 720 maturity group and can be utilized as main-crop silage hybrids in Bursa, Sakarya, and similar ecological regions. However, in both Sakarya and Bursa, medium-early silage hybrids are also used in addition to late-maturing ones. In Sakarya, medium-early silage hybrids are commonly grown as a second crop following potato or barley, while in Bursa, similar hybrids are cultivated after tomato and barley. As a main crop, both late and early-maturing hybrids are preferred. The preference for earlier hybrids in Sakarya is mainly due to the prevalence of intensive winter vegetable production. Likewise, in Bursa, where vegetable cultivation is also intensive, second-crop silage maize is widely cultivated on land that becomes available after the mid-summer harvest of processing tomatoes. Therefore, there is also a demand for earlier-maturing hybrids in these regions. Furthermore, with changing climatic conditions, maize cultivation areas have expanded to many parts of the country, and maize is now being grown even in provinces that were previously unsuitable due to cold conditions (*e.g*., Muş, Sivas). In such regions, hybrids with lower FAO maturity groups are considered more appropriate. Consequently, when initiating future silage maize breeding programs in Türkiye, it will be important to focus on earlier maturity groups, particularly within the FAO 400–600 range.

## Conclusions

The findings of this study demonstrated that the findings revealed that the S6 and S9 genotypes exhibited the highest forage yields, while S6 stood out with superior nutritional composition. Generally, DH-derived hybrids significantly outperformed the best-performing commercial checks, demonstrating the potential of the DH technology to introduce high-yielding and nutritionally superior hybrids into the market rapidly. The availability of high-quality forage to meet the nutritional needs of livestock is crucial for cost-effective and sustainable production. The superior hybrids developed in this study are planned to be included in the registration process in the near future. Following official variety trials, the release of these developed hybrids into the market is expected to contribute to closing the feed gap in the livestock sector.

## Supplemental Information

10.7717/peerj.20197/supp-1 Supplemental Information 1 Raw data. Maize genotypes for forage yield (t ha^-1^), plant height (cm), ear ratio (%), stem ratio (%), DM (%), CP (%), NDF (%), ADF (%), ADL (%), and RFV parameters.

## Additional Information and Declarations

### Data Availability

The following information was supplied regarding data availability:

The raw data is available in the Supplemental File.
